# Annotations

**Published:** 1894-09-01

**Authors:** 


### ANNOTATIONS.

### Mental Convalescents.

Medical science is widening its field of influence and authority. This is so because medical men are filling their calling with an ever-increasing sense of responsibility. They recognise and acknowledge the moral and social influence which they can bring to bear or withhold, as they choose, on the community, and alienists are now accepting the widest view of the extent of their field of labour. In former times, when a cure was effected the treatment ceased when the patient left the four walls of the asylum, and passed from the doctor's care. The prevention and predisposing causes of insanity and the condition of the convalescent were little studied. The convalescent from an attack of mania is now known to require as close attention and treatment as the convalescent from any other form of disease. This feeling is now wide spread, and at a recent meeting of the American Neurological Association, the after care of the insane received close and careful consideration. All those who entered into the discussion admitted the importance of the time of convalescence in the treatment of insanity, and it was generally held that patients usually leave the asylum or hospital too early. A suggestion new and important to America arose out of this conclusion. This was the establishment of convalescent homes for insane patients. Family surroundings and mismanagement often induce a relapse, and were such homes instituted, sufferers from melancholia especially might be removed from the hospital at an earlier date, and their recovery accelerated. These Homes have been tried successfully in England, Scotland, and France, and, as a result of the meeting, America too is likely to be better able to care for the insane in the stages of convalescence, a time when recovery or final insanity hangs in the balance. The history of crime, now so closely studied, has too frequently proved that a discharged patient from a madhouse, or from the guardianship of doctor and keeper, suffered not infrequently from a relapse to the danger of himself and his surroundings. A convalescent home should supply just the watchful care necessary without sharing the painful environments of the asylum.

### The Place of Soap in Strikes.

At present there is a strike going on in Scotland among the coal miners, and not only they, but the members of many trades which require large supplies of coal are idle. Meanwhile, wives and children beg or starve. They ask for food, as the primal necessity, but at least one woman, who came to the present writer's door, proffered a new request. Bread she had, at least enough for the day; but she said, "For mercy's sake give me a piece of soap. I've been married for twelve years and always managed to keep myself and my children clean; but now I haven't a penny to spare for soap," Such a demand sets one thinking on the fact that even the cleanliness which we demand of the poor as a condition of our help may sometimes be unattainable by them. Yet dirt means degradation. The dirty man feels himself separated as by a gulf from the more reputable-looking of his fellows, and instinctively withdraws into that more shady company (in every sense), where his griminess ceases to be remarkable. "Cleanliness is next to godliness," we say. Do we ever realise how much our godliness depends on cleanliness? Should we not be more lenient with the family that has dropped out of the habit of church going because they cannot present a decent appearance among their fellow-worshippers? Should we condemn so hastily the working woman whose kitchen presents a less spotless appearance than we, with soap, hot water, and scrubbing brushes galore, can attain to in our own? True it is that dirt, like other vices, is a thing which we "first endure, then pity, then embrace," but it is one of the vices into which deep poverty cannot help but fall. Let us try to cleanse our poor, and we shall put them on the first step of the ladder that leads to self-respect, independence, and health. Where there is no cleanliness, there must sooner or later be ill-health and disease also. Hence the well-being of all classes demands its general enforcement in large cities.

### Roof Gardens for Londoners.

London, taken as a whole, is a healthy city. The conditions, however, in which the larger portion of its inhabitants live cannot be termed hygienic, and the principal want is sufficient air. Much has already been done and is still being done by the gradual appropriation of open spaces as recreation grounds for the people. More might be accomplished if other employers would follow the example of the firm of city printers who have utilised their flat roofage as a recreation ground for their employés. These flat roofs are often of considerable extent in connection with large businesses, especially in the case of annexes, where a conical roof is often avoided to save obstruction of light. Not only in large buildings are these platforms wasted, but many small tenements have projections which are not accessible from any window, though they might easily have been so arranged, and a breath of air secured to the crowded inhabitants of the City. Neatly and securely railed, these little flat roofs can afford, and, indeed, in a certain number of cases, do afford, not only an aid to health, but permit of pleasure and occupation being found in the rearing of plants, and in the formation of hanging gardens. Many houses are so constructed that it is not possible to utilise the flat roof when it exists, but if those who are erecting houses in London would bear in mind the use to which any platform can be turned, they will increase the value of their property, and add a boon to their tenants too, by a little rearrangement of plans. There are few uglier sights than the backs of small London houses, which form a principal part of the scenery viewed from the railway carriages passing in and out of London. All this is capable of amelioration, and the traveller feels almost grateful when a little floriculture breaks the monotonous and gloomy outline. The poor need very little encouragement to take an interest in rearing plants, and in some streets a keen competition is visible. So beneficial a taste might be cultivated at very little cost, if the well-to-do on leaving town would remember their poorer neighbours to the extent of distributing the contents of window-boxes no longer required amongst them.

